# On Remittent Fever during August, 1839, in the Vicinity of Philadelphia

**Published:** 1839-09-21

**Authors:** Robert Burns

**Affiliations:** Frankford, Philadelphia county


### MEDICAL EXAMINER.

DEVOTED TO MEDICINE, SURGERY, AND THE COLLATERAL SCIENCES.

No. 38.] PHILADELPHIA, SATURDAY, SEPTEMBER 21, 1839. [Vol. II.

*On Remittent Fever during August*, 1839, *in the Vicinity of Philadelphia.* By Robert Burns, M. D.

To the Editors of the Medical Examiner.

Gentlemen,—In the 34th number, vol. 2d, of your journal, my attention was particularly directed to an article upon "the character of the diseases of the present season, at Philadelphia;" which afforded me at that time much satisfaction, inasmuch as I had then under my care eight cases of remitting fever, resembling very closely in their general character the description given in that article, and the course of treatment pursued was also somewhat similar.

In compliance, therefore, with the invitation given in the last sentence of said article, I take the liberty (although not heretofore having the privilege of being a correspondent) to give you "the results of my experience on this subject;" and I endeavour to do this the more willingly, because I view it as an imperative duty we owe to the profession and our fellow men generally, to make common stock of our experience through a proper channel, that nothing valuable, or in any degree useful, may be buried in forgetfulness.

Many important facts which would have enriched medical science,and enlarged to the community the resources and blessings of the healing art, have, through withholding a little labour, been lost; which may be compared to the treasures of a rich and productive mine, that after being safely raised above ground by much patient and persevering labour, are again thrown down, to be brought forth once more by a like expenditure of time and exertion. Being well assured that nothing very new or valuable will be found in what follows, I leave this communication to your judicious disposal.

Seven of the cases alluded to occurred among a population of forty-nine individuals, residing in ten houses, which are built upon a hill of considerable eminence, and surrounded by well cultivated fields. This location is situate about one mile from the river Delaware, and six from Philadelphia, on the Bristol turnpike. The eighth case was distant from the others about half a mile.

The first was that of a very old man, who had been indisposed for a considerable length of time, and did not consent to have medical aid until his strength so far failed him that he was unable to move from his bed. When visited he was in a state of extreme prostration, his pulse feeble and fluttering, his tongue and teeth were covered with a thick, brownish coating, his skin was hot and dry; there was also much restlessness and jactitation, with tremour of the hands; but his mental faculties were in their wonted vigour, and he calmly looked forward to the hour of his dissolution. In this case there was no hope of success, and all that could be done was to render him as comfortable as possible; for this purpose, he had a saline and anodyne mixture, with a gentle laxative. On the second day he died.

The second case was Mrs. S——, daughter of the above, about forty years of age, residing in the same house. She complained of headach, great soreness and lassitude, pains in the right side and back, constipated bowels, and much prostration; her fever was of a low character, and circulation feeble. Bloodletting appeared in this case out of the question; my object was to promote the biliary secretion, and evacuate the intestines, and thereby relieve the internal congestion, which evidently appeared to exist; for this purpose, the following was prescribed:

℞. Pil. Hydrarg. ℈i.
Pulv. R. Rhei ℈ii.
Ipecac. gr. iii.
Ant. Tart. et Potass. gr. i.
M. ft. mass. et div. in pilulas xx. S. Una, mane, meridie, et nocte.

Sinapism to right hypochondrium; pediluvia of salt and water, as hot as possible, at bedtime; light, farinaceous articles for food, and soda powders, or lemonade, for drink. By this course of treatment, in a few days convalescence ensued. Several dark and offensive dejections having been passed, by the use of chamomile and serpentaria, she was speedily restored to health.

The third case was a married female, mother of three children. She was seized with a chill, severe cephalalgia, pains in the back and extremities, sickness of stomach, pain in the right side, giving rise to troublesome spasmodic cough; although the fever in this case was active, and the burning heat of the skin very great, yet such was the feeble state of the pulse, that bloodletting was inadmissible. The conjunctiva was highly icterose, the complexion sallow, breathing frequent, the tongue heavily coated, with a thick, light brown fur, dark in the centre, but tip and edges red, bowels costive. From every symptom in this case, a decided course of treatment was demanded, consequently I commenced with the administration of the following purgative:

℞. Hyd. Chlor. Mit. gr. x.
Ext. Colocynth. Comp. gr. iii.
Pulv. Ipecac. gr. i. M. ft. pulv. i.

To be followed, if necessary, by a table-spoonful of the subjoined powder:

℞. Magnes. Ust.

Lac. Sulphuris $\overline{aa}$ ℥ss.

Ol. Cinnam. M. i. M. ft. pulv.

This treatment was followed by several dark, offensive dejections, with temporary amendment.

The headach, fever, sickness, and pain of right side, continued. With a view to equalize the circulation, aud act still further on the bowels, the following pills were prescribed:

℞. Pil. Hydrarg.

Pulv. R. Rhei āā ℈j.

G. Aloes ℈ss.

Pulv. Ipecac. gr. i.

Antim. Tart. gr. ss. M. ft. pil. x.

One of these was ordered to be taken every hour, until they operated freely on the bowels. These, with a saline mixture, had the happiest effect; for, at my next visit, the whole surface of the body was moistened with a warm perspiration, and a free discharge of dark-coloured fæces had taken place; the tongue was more moist, and beginning to clean off;—still, notwithstanding the great improvement in other respects, the sickness of stomach and pain of side remained, but these were effectually removed by a blister to the epigastrium, extending partly over the liver; the sallowness of the skin, with the yellow tinge of the eyes, speedily removed,—and, by the use of cinchona, serpentaria, and colombo, in infusion, at the expiration of ten days she was able to attend to her household affairs, although much enfeebled by the attack. The regimen and drinks used were similar to the preceding case. Duration of treatment from the 9th to the 20th of August.

The fourth, fifth, and sixth, were a mother and two of her daughters. These cases were more severe, and longer protracted than the preceding ones. In all three the remissions were of very short duration for eight or ten days, and the fever was characterized by a very severe degree of burning, particularly in the extremities; the skin was very dry, and produced to the touch an unpleasant, penetrating heat, which cannot be described by words more fitly than by the Latin phrase, *calor mordax.*

Mrs. L—— had much sickness of stomach, yellowness of the eyes, general sallow hue, feeble, slow pulse, and dark, scanty alvine discharges. The general treatment consisted of diaphoretics, the use of the following pill three times a day:

℞. Hyd. Chlor. Mit. gr. xii.

Ext. Colocynth. Comp. ʒss.

Pulv. R. Rhei ʒi.

Ipecac. gr. i. M. ft. pil. xii.

Farinaceous diet, ripe fruits, especially peaches, gum water, lemonade, and Seidlitz powders, for drink. The local treatment consisted of cups to the epigastrium, followed by an epispastic; sinapisms to the extremities, the application of cool water to the hands, arms, head, and face, which afforded much relief, and reduced the intolerable heat; when the remissions were such as to permit it, quinine was administered with much benefit.

The two daughters of this lady were affected in a similar manner,—but *gastritis* being a complication in one of them, the case assumed a very serious character. The prostration was very great, the skin hotter than the others, the same peculiar hue of complexion and eyes, much wildness of expression, the brain, however, unaffected; the tongue of a fiery redness, very smooth and glossy, but chapped in the centre; much sickness and pain at stomach. Treatment consisted of

Hyd. Chlor. Mit. gr. ss.
Pulv. Ipecac. Comp. gr. i.
Sacch. Alb. gr. ii. M. ft. pulv.

repeated every two hours; mucilaginous drinks, especially of the ulmus fulva; sinapisms to the epigastrium, followed by a blister; dressed with

Ungt. Hydr. Mitissimum, and the bowels kept open by mild laxatives. By this course, recovery advanced rapidly; and after the glassiness of the tongue had passed off, the use of quinine had a salutary effect.

The sister of this patient, who was younger, had a milder attack, but of longer continuance. By the use of spt. mindureri, small doses of calomel, mild laxatives, mucilaginous drinks and diet, with sinapisms, the case terminated well. These three cases, collectively, occupied from 9th to the 31st of August, inclusive, in their treatment.

The seventh case was that of an athletic young man. He was taken with a chill, followed by active fever, headach, universal soreness and lassitude; heat of skin not so great as in the other cases; conjunctiva of the same yellow hue; tongue thickly coated, dark in the centre; dejections scanty and dark coloured. The treatment was commenced by the following cathartic:

℞. Hyd. Chlor. Mit. gr. x.
Ext. Colocynth. Comp. gr. v.
Pulv. Ipecac. gr. i. M. ft. pulv.

followed by infusion of senna. This operated freely, bringing away dark, offensive stools,—the remissions in this case being so complete, that a weak tonic infusion was administered; but, at this time, it was not attended with any permanent success, the disease not being sufficiently overcome. The following mixture was prescribed to be taken during the exacerbation:

℞. Acid. Acetic. Comm. ℥ii.
Carb. Ammoniæ, q. s. ad sat.
Aq. Fontanæ ℥iv.
Antim. Tart. gr. ii.
Spt. Eth. Nit. f℥ss.
Tinct. Opii Camph. fʒi.
M. ft. Mix. S. Coch. Mag. q. h.

The bowels were kept open by the following pills:

℞. Mass. Hydr. gr. xii.
Ext. Colocynth. gr. xxiv.
Pulv. R. Rhei gr. xii.
Ext. Juglandis Cin. q. s. ad facien. pil. xii.
Sig. Capt. una ter in die.

By this course, a free diaphoresis was kept up, and the biliary secretion promoted; the tongue gradually became clean, and the sulphate of quinine completed the treatment, which occupied from the 18th to the 25th of August.

The eighth and last case with which I will trouble you at this time, was that of a little girl, about seven years of age, which I saw for the first time on the 15th of August. She had been sick for a week previous to my visit; and so high was her fever, and such her extreme restlessness, with an active, full pulse, that I was induced, under existing circumstances, to draw a little blood from the arm,—and although the quantity was small, it made a considerable impression upon the system. She had a troublesome cough, attended with pain, which she referred to the breast, the sequela of *pertussis;* the tongue was red on the tip and edges, with a whitish, speckled centre; there was extreme (restlessness,) fretfulness, and the countenance had a constant frown. The treatment was commenced with a cathartic of calomel et pulv. rhei, after which the following powders and mixture:

℞. Hyd. Chlor. Mit. gr. vi.

Pulv. R. Ipecacuanhæ gr. ii.

Opii gr. ss.

Sacch. Alb. ℈ss.

M. ft. pulv. xii. Sig. one every two hours.

℞. Acid. Citric. ʒi.

Carb. Potass. q. s.

Aq. Font. ℥iii.

Vin. Ipecac.

Tinct. Opii Camph. āā ♏ xx. M. ft. Mix.

a teaspoonful to be taken every hour when feverish; a blister to the chest and epigastrium; cool drinks; light gruel diet, with gum water, &c., constituted the principal remediate measures, under which the fever gradually subsided; and at the expiration of three weeks, the treatment, with the following tonics:

℞. Sulph. Quin. gr. iii.
Pulv. Rad. Aristol. Serp. gr. xii.
M. ft. p. pulv. xii. Sig. one every two hours during the intermission.

This quantity produced some sickness; and half a powder was given at a time, which produced no unpleasant effects, and speedily brought about convalescence. In this patient, slight deafness remained for a few days after the subsidence of the fever.

In concluding these remarks, the following inferences may be deduced from the preceding cases:

1st. Remitting fever, in this neighbourhood, differed from its usual character, in being marked with much prostration,—feeble and slow pulse, which would not warrant the employment of bloodletting,—and in closely resembling the milder grade of typhus.

2d. The combination of blue pill, colocynth., and ipecac., had a very happy effect in promoting the hepatic secretion, and evacuating the intestines. The good effects were still more apparent if slight nausea was produced, or the mercurial could be merely be perceived to act constitutionally.

3d. Gastric and hepatic congestion appeared to be prominent features in each case, which were promptly relieved by cups, sinapisms, blisters, and ipecacuanha.

4th. The intolerable burning of the skin was very peculiar, resembling, to the touch, the skin of a patient in scarlatina. This was much mitigated by cool bathing, and the diaphoretics mentioned above.

5th. When the tongue became moist, and began to clean off, the use of tonics, especially of quinine, at this time had a salutary effect, given during the remissions or intermissions, and recovery was generally speedy and permanent.

*Frankford, Philadelphia county*, Sept. 18, 1839.

